# The impact of childhood pneumococcal vaccination on hospital admissions in England: a whole population observational study

**DOI:** 10.1186/s12879-019-4119-8

**Published:** 2019-06-10

**Authors:** Tinevimbo Shiri, Noel D. McCarthy, Stavros Petrou

**Affiliations:** 1 Liverpool School of Tropical Medicine, International Public Health, Pembroke Place, L3 5QA, Liverpool, UK; 2 Warwick Clinical Trials Unit, Warwick Medical School, University of Warwick, Gibbet Hill Road, Coventry, CV4 7AL UK; 3 Population Evidence and Technologies, Warwick Medical School, University of Warwick, Gibbet Hill Road, Coventry, CV4 7AL UK

**Keywords:** Hospital admissions, Interrupted time series analysis, Pneumococcal conjugate vaccines, Pneumococcal disease, Indirect effects

## Abstract

**Background:**

Pneumococcal infections are major causes of morbidity and mortality worldwide. We use routine hospital admissions data and time-series modelling analysis to estimate the impact of the seven and thirteen valent pneumococcal conjugate vaccines (PCV7 and PCV13) on hospital admissions due to pneumococcal disease in England.

**Methods:**

Hospital admissions for pneumococcal meningitis, bacteraemia and pneumonia between January 1, 2003 and December 31, 2015 were identified from the national Hospital Episode Statistics database for all age groups in England. We model the impact of pneumococcal vaccination using interrupted time series analysis. Hospital admissions prior to vaccine introduction were extrapolated to predict the expected number of admissions in the absence of pneumococcal vaccines. Admissions avoided over time were estimated by comparing the fitted interrupted time series and the expected model for no vaccination in a Bayesian framework.

**Results:**

Overall, there were 43,531 (95% credible interval (CrI): 36486–51,346) fewer hospital admissions due to bacteraemia, meningitis and pneumonia in England during the period from 2006 to 2015 than would have been expected if pneumococcal vaccines had not been implemented, with the majority of hospital admissions avoided due to pneumonia. Among young children reductions in meningitis were more common, while among adults reductions in pneumonia admissions were relatively more important, with no evidence for reduced bacteraemia and meningitis among older adults. We estimated that 981 (95% CrI: 391–2018), 749 (95% CrI: 295–1442) and 1464 (95% CrI: 793–2522) bacteraemia, meningitis and pneumonia related hospital admissions, respectively, were averted in children < 2 years of age.

**Conclusions:**

Substantial reductions in hospital admissions for bacteraemia, meningitis and pneumonia in England were estimated after the introduction of childhood vaccination, with indirect effects being responsible for most of the hospital admissions avoided.

**Electronic supplementary material:**

The online version of this article (10.1186/s12879-019-4119-8) contains supplementary material, which is available to authorized users.

## Background

Pneumococcal infections are a major cause of long-term morbidity and mortality [1–3]. The introduction of childhood pneumococcal vaccination has resulted in significant reductions in pneumococcal disease in both vaccinated and unvaccinated individuals worldwide. Surveillance programmes on pneumococcal disease have shown that invasive pneumococcal disease due to serotypes covered by the vaccine have continued to decline following vaccine introduction [4–10]. However, the vaccine programme has also been associated with concomitant increases in carriage and disease due to other serotypes not contained in the vaccine [11–13]. There are concerns that serotype replacement could erode the benefits of the pneumococcal vaccine in the long term.

England and Wales introduced the seven-valent conjugate vaccine (PCV7; containing serotypes 4, 6B, 9 V, 14, 18C, 19F and 23F) in September 2006 as part of their universal paediatric immunisation programme, using a primary series of two doses at 2 and 4 months and a booster dose after the first birthday. During the first year of the programme, a catch-up campaign targeting children under 2 years was also implemented. In April 2010, PCV7 was replaced by a thirteen-valent vaccine (PCV13; containing the seven serotypes contained in PCV7 plus additional six serotypes 1, 3, 5, 6A, 7F and 19A) in the childhood vaccination programme. Vaccination coverage for the primary series and booster dose has risen from 80 and 83% in the first year of vaccine introduction to 90 and 94% in 2015/16, respectively [11, 12, 14]. Adults aged 65 years and at-risk groups aged 2 years or over are typically given the polysaccharide pneumococcal vaccine (PPV23; containing serotypes 1, 2, 3, 4, 5, 6B, 7F, 8, 9 N, 9 V, 10A, 11A, 12F, 14, 15B, 17F, 18C, 19A, 19F, 20, 22F, 23F, and 33F). PPV23 immunisation in adults was introduced in August 2003. During the first year, all people ≥80 years were eligible for a single dose and in April 2004 it was extended to cover people aged ≥75 years. In April 2005, a universal PPV23 schedule for adults aged ≥65 years was introduced and coverage in this age group has remained stable at around 70% since introduction [15]. PCV is not available for adults in England and Wales apart from the severely immunocompromised.

Although studies using laboratory-based surveillance data have shown sustained decreases in invasive pneumococcal disease for the whole population in England and Wales since the introduction of childhood vaccination programmes [11, 12], the use of microbiologically confirmed cases might under estimate the burden of disease as they focus on invasive disease confirmed by culture from sterile sites and not on culture negative pneumonia. There is limited data on the impact of vaccination on long-term trends in hospital admissions following vaccine introduction for individual pneumococcal disease infections [16, 17]. Improved knowledge of disease burden is critical to inform future research and guide intervention policy [18–20]. We assessed the impact of pneumococcal vaccination on individual and composite pneumococcal disease hospitalisation in England from 2003 to 2015 using interrupted time series analysis.

## Methods

### Datasets

Data was extracted from the Hospital Episode Statistics (HES) database, which contains information on all acute care hospitalisations in England. Datasets analysed comprised of all inpatient hospitalisations between January 1, 2003 and December 31, 2015. Cases were identified by the presence of relevant diagnostic discharge codes (primary or secondary diagnosis) listed in the patient’s discharge record. The discrete health states or medical conditions associated with *Streptococcus pneumoniae* infection identified were bacteraemia (International Classification of Diseases, 10th revision [ICD-10], code A403), meningitis (G001) and pneumonia with or without a positive culture from a sterile site (J13). Our data represents more severe cases of pneumococcal infections requiring hospitalisation.

The number of inpatient hospitalisations attributed to pneumococcal infections were aggregated by month and stratified according to the following age groups: < 2 years, 2–4 years, 5–14 years, 15–44 years, 45–64 years, and ≥ 65 years. Although all hospital episodes for each patient were obtained, we only used the first admission episode that was due to a pneumococcal infection for each patient in any epidemiological year. The period January 2003 to March 2007 was defined as the pre-PCV7 period, April 2007 to October 2010 as the PCV7-era or pre-PCV13 period and November 2010 onwards as the post-PCV13 period. Data on mid-year and age-specific national population estimates from 2003 to 2015 was obtained from the Office for National Statistics, the national statistical institute of the United Kingdom.

### Statistical analysis

For each disease category, we ran age-specific Poisson regression models with and without vaccination on monthly time series. We fitted segmented Poisson models using a Bayesian framework. Uncertainty was given by 95% credible intervals (CrIs) obtained using the parameter posterior distributions. We compared the regression models without the impact of vaccination with those with the impact of vaccination (i.e., the interrupted time series regression) to test whether there was a change over time and step change of hospital admissions after vaccine introduction (see Additional file 1). In the interrupted time series model, we created an indicator variable taking the value 1 for the PCV7 era and 0 for both the pre-PCV7 and the period after switching PCV7 with PCV13. The second indicator variable captured the pre-PCV13 and post-PCV13 periods. The expected trend in the absence of any vaccination (the counterfactual) was based on the pre-PCV7 data. Cases (hospital admissions) averted over time were calculated by comparing the fitted interrupted time series and the counterfactual model. We used the results of these calculations to estimate cumulative reductions in hospital admissions.

Our pre-vaccine data covers a short period, and we extrapolated the pre-vaccine trend based on 3 years data out for approximately 10 years. Because this might give unreliable estimates we tested the robustness of our analysis by also fitting the counterfactual model taking up to April 2010 as baseline. We also fitted a series of regression models changing the lag period before any PCV7-vaccine impact is expected. We varied this period from January 2007 to October 2007. In addition, as well as considering the two intervention schedules, PCV7 and PCV13, separately, we also conducted analyses assuming that the impact of PCV13 was similar to that of PCV7.

## Results

During the years studied, January 1, 2003 to December 31, 2015, there were 60,989 cases of patients with a first admission for any of the three pneumococcal diseases studied, with 11,617 (19%), 6062 (10%), and 43,310 (71%) cases of bacteraemia, meningitis, and pneumonia, respectively. Hospital admissions showed seasonal fluctuations with peaks occurring in winter months. Monthly hospitalisations for bacteraemia and meningitis were lowest in children 2–14 years of age.

### Interrupted time series analyses

**Fig. 1:**
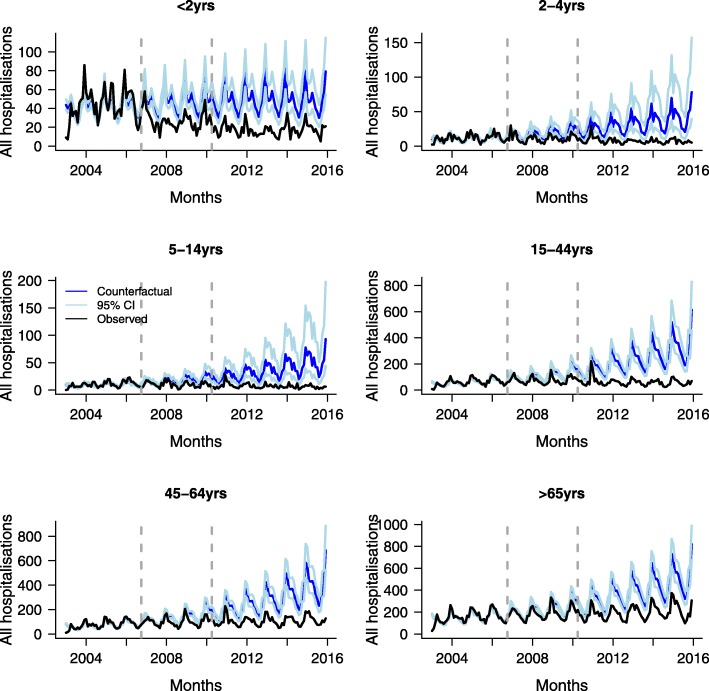
Observed and fitted monthly trends in all pneumococcal disease -related hospitalisations between 2003 and 2015 by age category. Vertical grey lines represent the months when PCV7 and PCV13 were introduced into the immunisation programme, respectively. Counterfactual – expected hospital admissions in the absence of vaccination (blue lines). 95% credible intervals are given in light blue lines

**Fig. 2:**
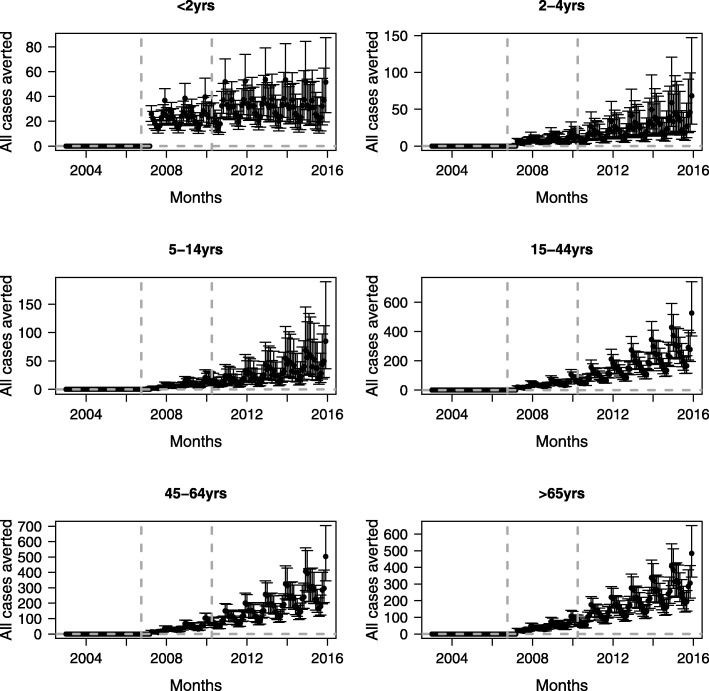
Estimated monthly pneumococcal disease admissions averted due to the introduction of the vaccination programmes by age category. Error bars represent the 95% credible intervals. Vertical lines represent the months when PCV7 and PCV13 were introduced into the immunisation programme, respectively

In Table 1 and Figs. 1 and 2, we present observed and model-predicted hospital admissions, and the number of hospital admissions avoided by disease and age category. Reductions in hospitalisations for all studied pneumococcal diseases combined were observed in all age groups. In children < 2 years, 2–4 years and 5–14 years, the number of admissions averted during the study period were 3001 (95% CrI: 1987–4314), 2026 (95% CrI: 972–3809) and 2302 (95% CrI: 1058–4509), respectively (Table 1). The number of admissions averted for adults 15–44 years, 45–64 years and ≥ 65 years were 13,533 (95% CrI:9822–18,214), 13,719 (95% CrI: 9628–18,556) and 14,909 (95% CrI: 10693–19,564), respectively.

**Table 1:** Observed and estimated (in the presence and absence of vaccination) total number of hospital admissions and admissions averted between 2003 and 2015, including 95% credible intervals^a^

	Bacteraemia	Meningitis	Pneumonia	All diseases
Age group				
< 2 years				
Observed	1248	1818	1243	4309
ITS^b^	1248 (1180, 1318)	1818(1735, 1903)	1243(1176, 1314)	4308(4181, 4440)
Counterfactual^c^	2232 (1632, 3269)	2566(2103, 3265)	2707(2030, 3770)	7309(6276, 8629)
Admissions averted^d^	−981(− 2018, − 391)	− 749 (− 1442, − 295)	−1464(− 2522, − 793)	−3001(− 4314, − 1987)
2–4 years				
Observed	411	227	861	1499
ITS	411 (372, 452)	227(198, 257)	861(804, 920)	1499(1423, 1575)
Counterfactual	536 (322, 1078)	607(268, 1753)	2595(1542, 4740)	3525(2463, 5325)
Admissions averted	−126(− 661, + 87)	− 379(− 1526, − 45)	− 1734(− 3871, − 692)	−2026(− 3809, − 972)
5–14 years				
Observed	290	279	796	1365
ITS	290 (257, 324)	279(248, 313)	795(741, 852)	1365(1294, 1439)
Counterfactual	703 (273, 2321)	647(295, 1778)	2407(1438, 4346)	3667(2414, 5884)
Admissions averted	−413(− 2030, + 14)	−368(− 1492, − 19)	− 1611(− 3536, − 647)	−2302(− 4509, − 1058)
15–44 years				
Observed	1546	939	7481	9966
ITS	1545(1469, 1624)	939(880, 1000)	7480(7312, 7652)	9967(9772, 10,163)
Counterfactual	2557(1750, 3922)	1499(950, 2569)	20,285(16,507, 25,257)	23,497(19,788, 28,185)
Admissions averted	−1011(− 2374, − 212)	− 560(− 1625, − 12)	−12,798(− 17,750, − 9020)	−13,533(− 18,214, − 9822)
45–64 years				
Observed	2620	1549	10,826	14,995
ITS	2619(2521, 2722)	1549(1473, 1626)	10,827(10,628, 11,031)	14,994(14,755, 15,236)
Counterfactual	4772(3458, 6866)	1995(1399, 2996)	22,598(18,790, 27,438)	28,712(24,596, 33,567)
Admissions averted	−2102(− 4232, − 854)	−448(− 1442, + 141)	− 11,777(− 16,597, − 7979)	− 13,719(− 18,556, − 9628)
≥ 65 years				
Observed	5392	1199	21,781	6955
ITS	5392(5249, 5538)	1199(1133, 1267)	21,779(21,492, 22,070)	28,371(28,040, 28,708)
Counterfactual	4722 (3459, 6866)	1221(836, 1891)	38,751(34,403, 43,763)	43,284(39,036, 47,987)
Admissions averted	+ 20(− 1199, + 929)	−21(− 684, + 359)	− 16,963(− 21,937, − 12,662)	−14,909(− 19,564, − 10,693)
All age groups				
Observed	11,507	6011	42,988	60,506
ITS	11,506(11,298, 11,718)	6011(5862, 6165)	42,986(42,580, 43,393)	60,502(60,024, 60,979)
Counterfactual	15,080(13,171, 17,492)	8266(7123, 9697)	84,821(77,680, 92,722)	104,022(96921111847)
Admissions averted	− 3571(− 5973, − 1682)	− 2258(− 3667,-1122)	−41,845(− 49,706,-34,692)	−43,531(− 51,346,-36,486)

^a^For each diagnosis, separate models were fitted by age group

^b^ITS – interrupted time series - model the impact of vaccination

^c^Counterfactual – expected hospital admissions in the absence of vaccination

^d^Admissions averted since April 2007

When the analysis was stratified by disease, bacteraemia hospital admissions averted in age groups < 2 years, 15–44 years and 45–64 years decreased over time after the introduction of PCV7 (Table 1; Additional file 1: Figures. S1, S2 and S3 showing monthly trends, risk rate ratios and number of admissions averted, respectively). In these age groups, the total number of admissions averted over the study period were 981 (95% CrI: 391–2018), 1011 (95% CrI: 212–2374) and 2102 (95% CrI: 854–4232), respectively. In other age groups, no significant changes were observed in the number of hospital admissions due to bacteraemia.

A substantial number of meningitis-related hospital admissions were averted in children < 15 years, although in children 2–15 years significant reductions were only observed after switching PCV7 with PCV13 vaccine. An estimated total of 749 (95% CrI: 295–1442), 379 (95% CrI: 45–1526), 368 (95% CrI: 19–1492) and 560 (95% CrI: 12–1625) admissions were averted in children < 2 years, 2–4 years, 5–14 years and young adults 15–45 years, respectively (Table 1; Additional file 1: Figures S4, S5 and S6, showing monthly trends, risk rate ratios and number of admissions averted, respectively). There were no significant changes in the number of meningitis-related hospital admissions in older adults ≥45 years of age.

In all age groups, the number of pneumonia-related hospital admissions averted was substantial and sustained over time. (Table 1; Additional file 1: Figures S7, S8 and S9 showing monthly trends, risk rate ratios and number of admissions averted, respectively). These ranged from 1464 (95% CrI: 793–2522) in children < 2 years to 16,963 (95% CrI: 12662–21,937) in adults ≥65 years of age.

Overall, there were 43,531 (95% CrI: 36486–51,346) fewer hospital admissions in England when the three diseases are combined over the period of study than would have been expected if pneumococcal vaccines had not been implemented (Table 1; Additional file 1: Figures S10, S11 and S12 showing monthly trends, risk rate ratios and number of admissions averted, respectively). The large majority of these reductions (approximately 96%) were due to pneumonia hospital admissions and most of these reductions were in adult populations ≥15 years of age.

In a sensitivity analysis where we fitted the counterfactual model taking up to October 2010 as the PCV7-era and then switching off the impact of the vaccine, we could still reproduce counterfactual trends for no vaccine (Additional file 1: Figure S13). In addition, we observed consistent results when we assumed a similar vaccine effect for PCV7 and PCV13 (Additional file 1: Figure S14).

In Additional file 1: Figure S15 we present the effects of variation in lag period before any vaccine effect is expected (from January 2007 to October 2007). The results show that a longer delay to when the PCV7 effect is modelled results in a diminished role of the vaccination programmes. Overall, our results were robust to changes in the lag period before any vaccine is expected to have an impact, as demonstrated by the estimated risk ratios over time presented in Additional file 1: Figure S16.

## Discussion

In this study, we have assessed the impact of the introduction of pneumococcal conjugate vaccines in England on hospital admissions due to bacteraemia, meningitis, and pneumonia infections between January 2003 and December 2015. Our estimates suggest that the childhood pneumococcal vaccination programme was associated with substantial reductions in pneumococcal disease-related hospitalisations in all age groups, with indirect effects being responsible for most of the hospital admissions avoided. When stratified by disease condition, we observed a clear impact of vaccines in young children < 2 years for all pneumococcal infections. Non-significant reductions were observed for bacteraemia associated hospital admissions in children 2–4 years and 5–14 years of age. In the elderly ≥65 years of age, the vaccine indirect impact was diminished - with a trend towards an increase in bacteraemia hospitalisations. The small number of admissions observed in this age group might have limited our power to detect relatively small changes in the number of hospital admissions.

There was a sustained and substantial reduction in the number of hospital admissions due to meningitis in children < 15 years of age and young adults 15–44 years of age, whereas in older adults ≥65 years of age, there were no significant changes in the number of meningitis-related hospital admissions. These results are similar to those found in an analysis of hospital admissions data from 1999 to 2011 [21]. In children aged 2–4 years and 5–14 years, the number of hospital admissions due to meningitis did not change significantly after the introduction of PCV7 into the childhood immunisation programme. Plots of rate ratios over time before and after pneumococcal vaccines show a pattern of decline after PCV7 was replaced with PCV13 in 2010, similar to what has been observed using laboratory-based surveillance data [22]. These results mirror laboratory-based surveillance data from other settings where there was a marked decline in pneumococcal meningitis among children < 2 years and no significant change in older age groups [7, 23, 24].

Our results indicate that pneumococcal conjugate vaccines have prevented hospital admissions due to pneumonia, under which both invasive pneumococcal disease and non-invasive disease were recorded, in all age groups with 90% of reductions in individuals ≥15 years of age occurring through herd immunity. A broadly similar 90% reduction in pneumonia-related hospital admissions was observed in adults in the United States [25]. Despite these huge reductions, a substantial burden of pneumonia remains, particularly in adult age groups.

It has been demonstrated that pneumococcal vaccines have substantially reduced invasive pneumococcal disease in the whole population of England and Wales. Our data extend these observations to show significant reductions in hospital admissions in all age groups, as a result of clinically-diagnosed pneumonia that comprised the largest quantitative reduction in admissions. The patterns of decline in all hospitalisations due to the combined three pneumococcal diseases reflect those observed from the surveillance data based on laboratory confirmed IPD cases over the same period [11, 12, 14]. Including the latest data up to the epidemiological year 2016/17, Ladhani et al. estimated that paediatric vaccination approximately saved 40,000 cases of invasive pneumococcal disease [12]. This number is lower, although not significantly so, than our estimate of 43,531 (95% CrI: 36486–51,346), despite the surveillance data having more data points from an extra year and more data from Wales. Using HES data, we were able to conduct our estimation for individual disease outcomes, and further include combined disease outcomes.

Our study, using available national hospital data, does not cover recent years where preliminary laboratory surveillance data suggests significant replacement disease due to non-vaccine serotypes, particularly in adults. The recent increase in replacement disease therefore warrants further work using the methods we have used here as sufficient later data becomes available. Previous studies have shown an increase in admissions to hospital for pneumococcal disease before the introduction of pneumococcal vaccines [17, 21, 26]. In the Oxfordshire region of England, community acquired pneumonia hospitalisations from all causes have been increasing, with no evidence that this increase is influenced by coding practices or changes in population demographics [16]. The rise may be due to population factors, changes in health care organisation, biologic processes or a combination of these factors [17]. Given this background, in the absence of vaccines, hospitalisations due to pneumococcal diseases studied here are expected to have increased, as shown by our counterfactual trends.

Our study has several limitations. First, we did not have serotype-specific data, so the analysis could not separate reductions due to vaccine serotypes from non-vaccine serotype disease. Second, we have relied on administrative data that is based on diagnostic codes, which we have assumed were assigned correctly; this may not always be the case as there may be miscoding. Third, we did not attempt to infer the number of hospital admissions avoided due to the use of PPV23 in adults. However, the greatest reduction in burden was among adults under 65 years, rather than the age group receiving PPV23 routinely, so that the effect of PPV23 may have been small. Lastly, the interrupted time series used assumes that the pre-vaccination trends are linear and continue to be linear in the post vaccine period, thus missing unexpected effects that might affect the number of hospitalisations observed. We also did not have data on non-pneumonia hospitalisations that could have been useful to capture counterfactual trends in hospitalisations accurately.

Despite these limitations, our study covers the longest observation period post vaccine introduction of any analysis examining the impact of pneumococcal vaccinations on hospitalisation due to individual pneumococcal diseases such as bacteraemia, meningitis and pneumonia in England. Using ICD-coded data we have shown that vaccines have reduced the burden of pneumococcal disease-related hospital admissions in unvaccinated populations, particularly in young adults, similar results to those of laboratory-based surveillance. However, the burden of meningitis and bacteraemia has not changed in adults ≥65 years of age, despite this population receiving PPV23 in addition to indirect effects generated by the childhood programme. There may be utility in inferring the population-based impact of pneumococcal vaccines using health-care data in countries with little, or no laboratory-based surveillance for pneumococcal disease, including many low and middle-income countries.

## Conclusions

This study provides additional observational evidence about temporal trends in pneumococcal disease in England before and after the introduction of paediatric pneumococcal vaccinations. Our analysis has shown that these vaccines have significantly reduced the burden of pneumococcal disease related hospital admissions, including both culture confirmed and culture negative pneumonia, with indirect effects being responsible for most of the hospital admissions avoided.

## Additional file

Additional file 1: This supplementary file contains detailed statistical approaches used to analyse the data, additional results for individual disease conditions and sensitivity analysis exploring the impact of fitting different counterfactual models and adjusting the time when vaccines’ effect is started in the time series analysis (DOCX 747 kb)

## Data Availability

The data that support the findings of this study are available from the Department of Health, England, but restrictions apply to the availability of these data, which were used under license for the current study, and so are not publicly available. Data are however available from the authors upon reasonable request and with the permission of the Department of Health.

## Abbreviations

CrI: credible interval
HES: Hospital Episode Statistics
ICD: International Classification of Diseases
PCV13: Thirteen-valent conjugate vaccine
PCV7: Seven-valent conjugate vaccine
PPV23: Twenty-three valent polysaccharide pneumococcal vaccine
