# Supplementary material for: The impact of childhood pneumococcal vaccination on hospital admissions in England: a whole population observational study
Source: BMC Infect Dis. 2019 Jun 10;19:510. doi: 10.1186/s12879-019-4119-8 (PMC6558731; doi:10.1186/s12879-019-4119-8)
Supplement: Supplementary file 1 — This supplementary file contains detailed statistical approaches used to analyse the data, additional results for individual disease conditions and sensitivity analysis exploring the impact of fitting different counterfactual models and adjusting the time when vaccines’ effect is started in the time series analysis (DOCX 747 kb) [file 12879_2019_4119_MOESM1_ESM.docx]

**Additional file 1**

Descriptive analysis 2

Methods 2

Results 4

Bacteraemia 4

Meningitis 7

Pneumonia 10

Combined age groups 13

Sensitivity analysis 15

References 19

# Descriptive analysis

We present a summary of the average hospital admissions between January 1, 2003 and December 31, 2015 by age category in Table S1.

**Table S1.** Observed annual average hospital admissions between 2003 2015 by age category

|  | **Septicaemia** | **Meningitis** | **Pneumonia** | **All diseases** |
| --- | --- | --- | --- | --- |
|  | mean (range) | mean (range) | mean (range) | mean (range) |
| **Age group** |  |  |  |  |
| <2 years | 136 (112 – 148) | 227 (207 – 250) | 165 (124 – 190) | 527 (443 – 573) |
| 2-4 years | 36 (31 – 45) | 23 (18 – 31) | 82 (68 – 104) | 141 (118 – 170) |
| 5-14 years | 19 (14 – 23) | 25 (20 – 29) | 76 (45 – 94) | 119 (82 – 143) |
| 15-44 years | 118 (93 – 139) | 74 (62 – 87) | 536 (355 – 655) | 727 (510 – 872) |
| 45-64 years | 174 (129 – 214) | 107 (91 – 121) | 637 (396 – 765) | 918 (616 – 1 092) |
| ≥65 years | 360 (345 – 377) | 80 (68 – 93) | 1 429 (1 020 – 1 656) | 1 869 (1 449 – 2 088) |
| All age groups | 842 (741 – 913) | 535 (469 – 566) | 2 924 (2 008 3 410) | 4 301 (3 218 – 4 843) |

# Methods

We present the statistical analysis and approaches used to assess the impact of vaccination on hospital admissions.

The number of hospitalisations, $X_{i}$, was assumed to follow a Poisson distribution with mean $\mu_{i}$. Hospitalisations were modelled using segmented regression models. We compared the regression models without the impact of vaccination with the interrupted time series regression to test whether the slope and the level of the line changed after vaccine introduction. In the interrupted time series model, we created an indicator variable, $pcv7_{dum}$, taking the value 1 for the PCV7-era and 0 for both the pre-PCV7 and the period after switching PCV7 with PCV13. The second indicator variable, $pcv{13}_{dum}$, indicated the pre-PCV13 (coded 0) and post-PCV13 (coded 1 after April 2010). The segmented regression model is given by:

$${log(\mu}_{i})= \beta_{0}+\beta_{1}.t_{i}+\beta_{2}.pcv7_{dum}+\beta_{3}.t_{i}.pcv7_{dum}+\beta_{4}.pcv{13}_{dum}+ \beta_{5}.t_{i}.pcv{13}_{dum}+\sum_{j=6}^{16} \beta_{j}.\mathrm{month}_{j,i}+\log\left( N_{i} \right), (1)$$

where $\beta_{0}$ is the baseline number of cases at time 0, $\beta_{1}$ is the change in the number of hospitalisations associated with a time unit change, $\beta_{2}$ is the level change following the introduction of PCV7, $\beta_{3}$ indicates the slope change following the introduction of PCV7 (using the interaction between time and PCV7 intervention), $\beta_{4}$ is the level change following the introduction of PCV13, $\beta_{5}$ indicate the slope change following the introduction of PCV13 (using the interaction between time and PCV13 intervention) and $\beta_{j}(j=6,5,\ldots, 16)$ represents the impact of seasonality measuring the difference between the other months and January (i.e., $\mathrm{month}_{j,i}=1$ if $i$ is month $j$ and zero otherwise). $N_{i}$ is an offset term representing the age-standardised population (mid-year population estimates) to adjust for any potential changes in the population over time.

The expected trend in the absence of PCV13, i.e., the counterfactual for no PCV13 vaccine and stopping PCV7 is given by

$${log(\mu}_{i})= \beta_{0}+\beta_{1}.t_{i}+\beta_{2}.pcv7_{dum}+\beta_{3}.t_{i}.pcv7_{dum}+\sum_{j=6}^{16} \beta_{j}.\mathrm{month}_{j,i}+\log\left( N_{i} \right) (2)$$

The model is fitted to the pre-PCV7 and PCV7 vaccine era and then extrapolated to the post PCV13 to give the expected trend in the absence of PCV13 and stopping of PCV7.

The expected trend in the absence of any vaccination programme, i.e., the counterfactual for neither PCV7 nor PCV13 vaccine is given by

$${log(\mu}_{i})= \beta_{0}+\beta_{1}.t_{i}+\sum_{j=6}^{16} \beta_{j}.\mathrm{month}_{j,i}+\log\left( N_{i} \right) (3)$$

The model is fitted to the pre-vaccine period and then extrapolated to the post vaccine period to give the expected trend that would have been observed in the absence of vaccine interventions. In our base analysis, we assume that the impact of PCV7 is observed after six months of vaccine introduction, i.e., the pre-vaccine period spans the period between January 2003 and April 2007.

We implemented a Bayesian approach to the segmented Poisson models to assess the impact of pneumococcal vaccination over time. The following non-informative prior distributions were assigned: $\beta_{i}\sim\mathrm{Uniform}\left( -100, 100 \right), i=0,1,2,\ldots,16$. Posterior distributions were obtained through a Markov Chain Monte Carlo method, based on a chain of length 20 000, after a burn-in of 70 000 iterations. The models were fitted using JAGS ^1^ in the R software environment (version 3.3.1) ^2^. Separate models were fitted for separate age groups and all age groups combined, individual disease conditions and all disease cases combined. Uncertainty was given by 95% credible intervals obtained using the parameter posterior distributions.

We also fit a series of regression models by changing the lag period before any PCV7-vaccine impact is expected. We vary this time from October 2006 to July 2007.

In addition, rather than considering two interventions PCV7 and PCV13 separately, we assume that the impact of PCV13 was similar to that of PCV7, such that in our interrupted time series analysis we only introduce one dummy variable, ${pcv}_{dum}$, that is zero before the introduction of PCV7 and 1 elsewhere. The segmented regression model is given by:

$${log(\mu}_{i})= \beta_{0}+\beta_{1}.t_{i}+\beta_{2}.{pcv}_{dum}+\beta_{3}.t_{i}.{pcv}_{dum}+\sum_{j=6}^{16} \beta_{j}.\mathrm{month}_{j,i}+\log\left( N_{i} \right), (4)$$

with the counterfactual for no vaccination being given by equation 3.

# Results

We present model fits for individual pneumococcal diseases in Figures S1 to S12.

### Bacteraemia

The median time trend, rate ratios over time and admission cases avoided for septicaemia are shown in Figure S1, S2 and S3, respectively.


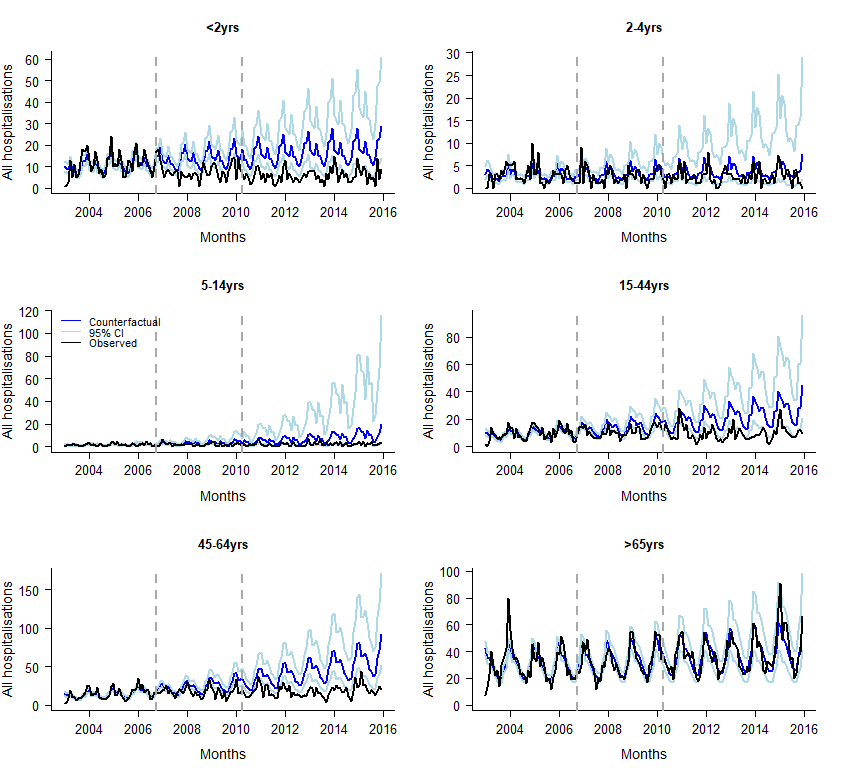


**Figure S1**. Observed and fitted monthly trends in bacteraemia hospitalisations between 2003 and 2015 by age category. Vertical grey lines represent the months when PCV7 and PCV13 were introduced into the immunisation programme, respectively.


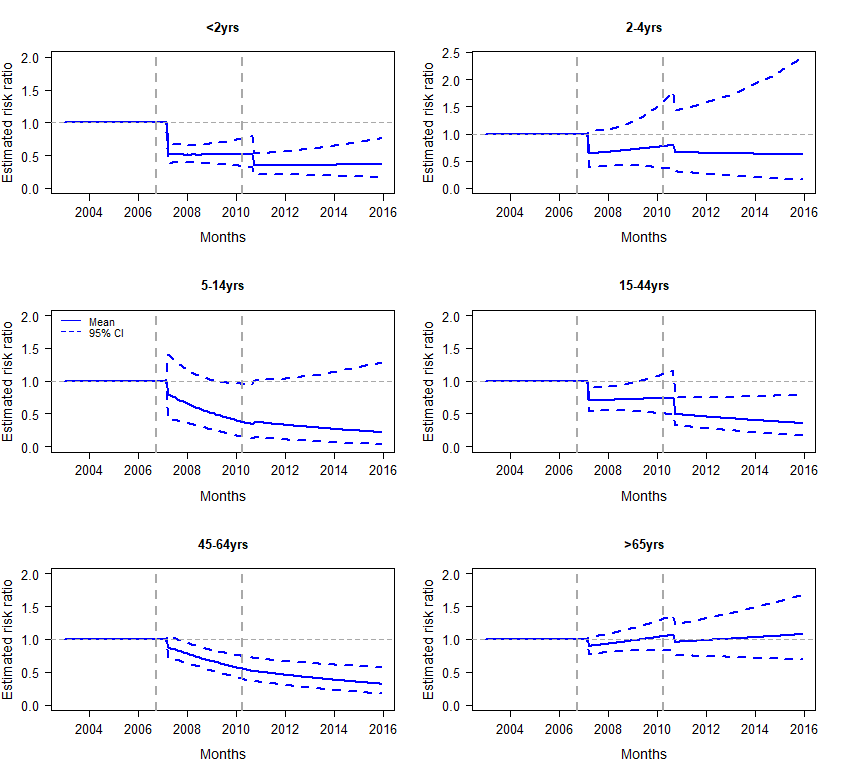


**Figure S2**. Bacteraemia monthly hospitalisation rate ratios over time by age category. Dashed lines represent the 95% credible intervals. Vertical grey lines represent the months when PCV7 and PCV13 were introduced into the immunisation programme, respectively.


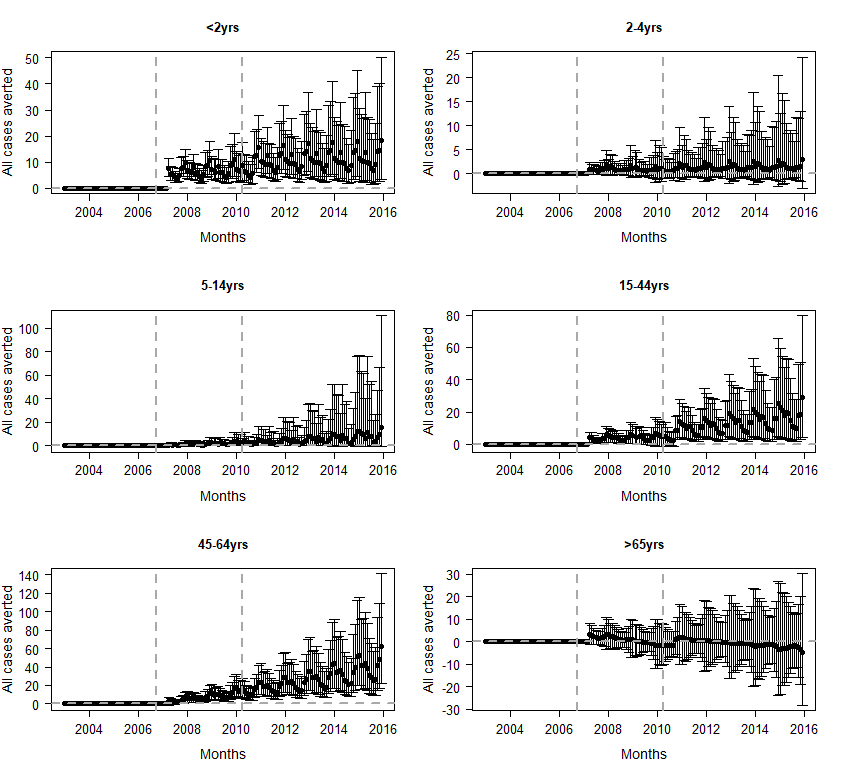
**Figure S3**. Estimated monthly bacteraemia admissions averted due to the introduction of the vaccination programmes by age category. Error bars represent the 95% credible intervals. Vertical lines represent the months when PCV7 and PCV13 were introduced into the immunisation programme, respectively.

### Meningitis

The median time trend, rate ratios over time and admission cases avoided for meningitis are shown in Figure S4, S5 and S6, respectively.


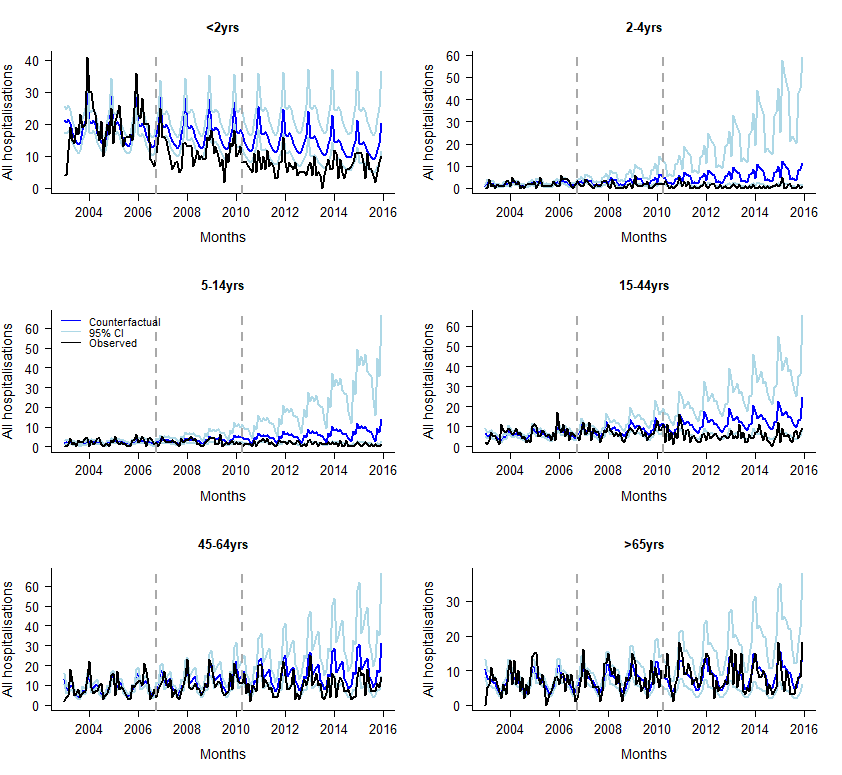


**Figure S4**. Observed and fitted monthly trends in meningitis hospitalisations between 2003 and 2015 by age category. Vertical grey lines represent the months when PCV7 and PCV13 were introduced into the immunisation programme, respectively.


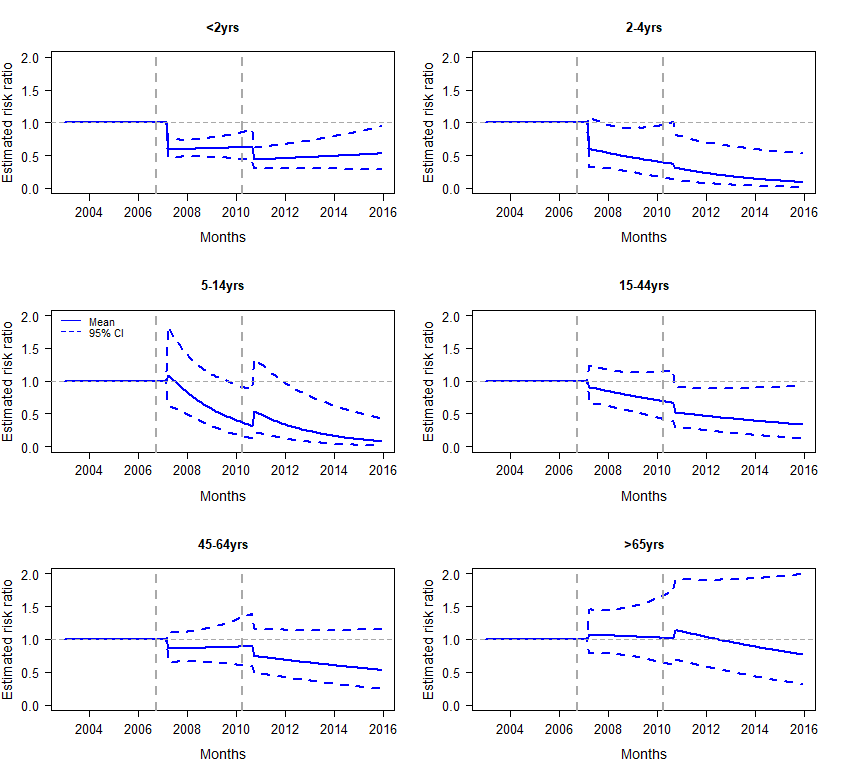


**Figure S5**. Meningitis monthly hospitalisation rate ratios over time by age category. Dashed lines represent the 95% credible intervals. Vertical grey lines represent the months when PCV7 and PCV13 were introduced into the immunisation programme, respectively.


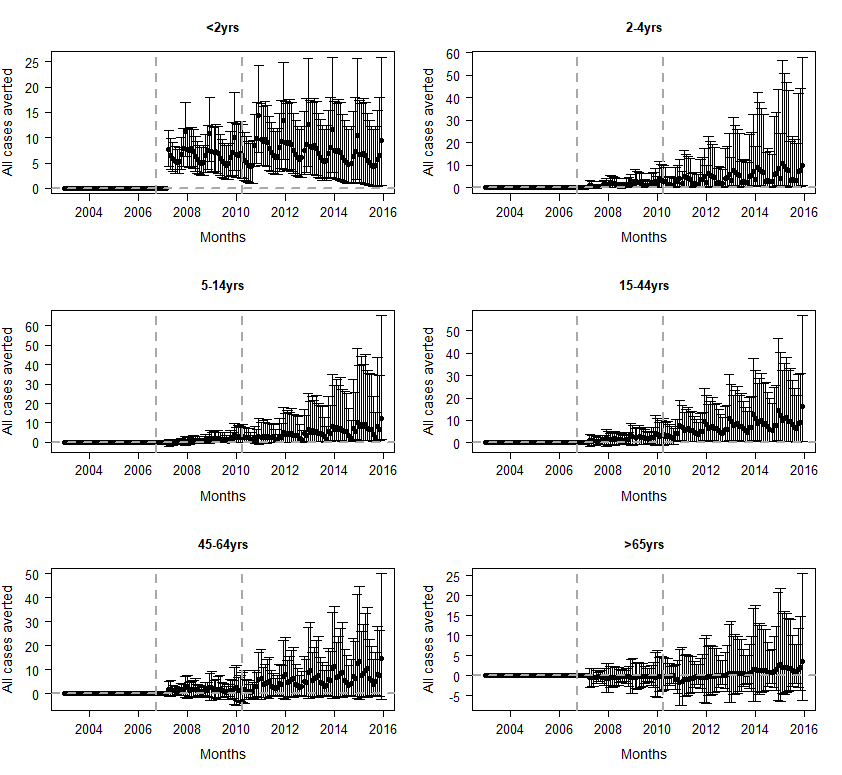


**Figure S6**. Estimated monthly meningitis admissions averted due to the introduction of the vaccination programmes by age category. Error bars represent the 95% credible intervals. Vertical lines represent the months when PCV7 and PCV13 were introduced into the immunisation programme, respectively.

### Pneumonia

The median time trend, rate ratios over time and admission cases avoided for pneumonia are shown in Figure S7, S8 and S9, respectively.


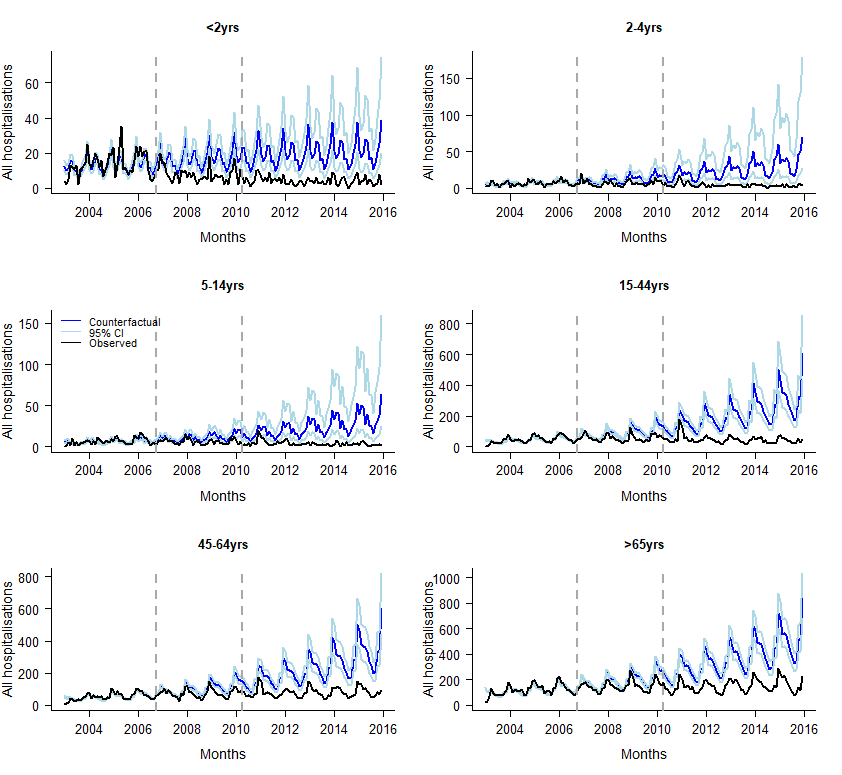


**Figure S7**. Observed and fitted monthly trends in pneumonia hospitalisations between 2003 and 2015 by age category. Vertical grey lines represent the months when PCV7 and PCV13 were introduced into the immunisation programme, respectively.


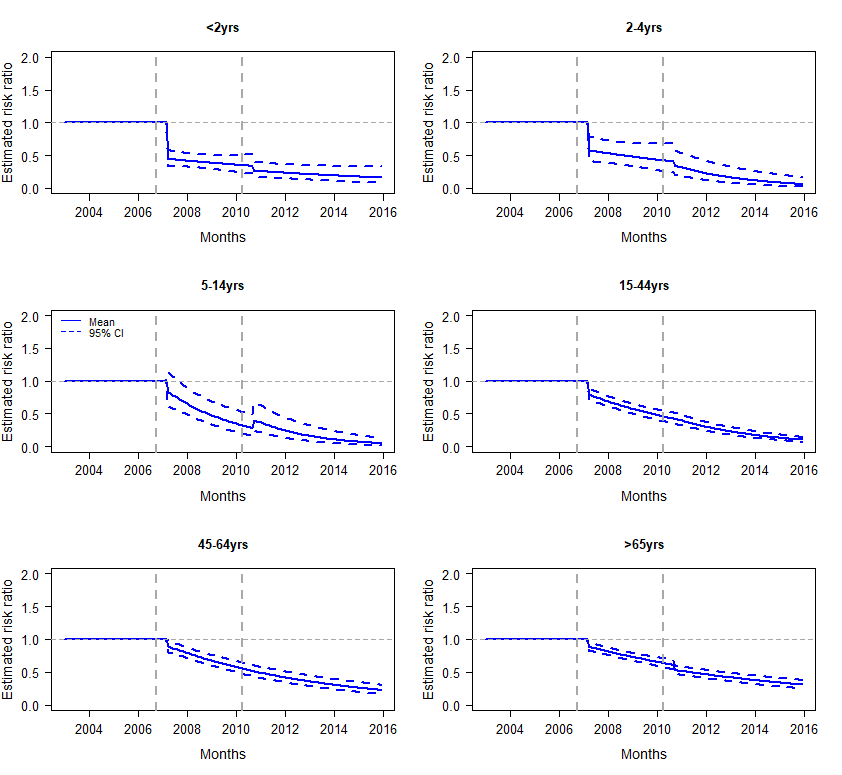


**Figure S8**. Pneumonia monthly hospitalisation rate ratios over time by age category. Dashed lines represent the 95% credible intervals. Vertical grey lines represent the months when PCV7 and PCV13 were introduced into the immunisation programme, respectively.


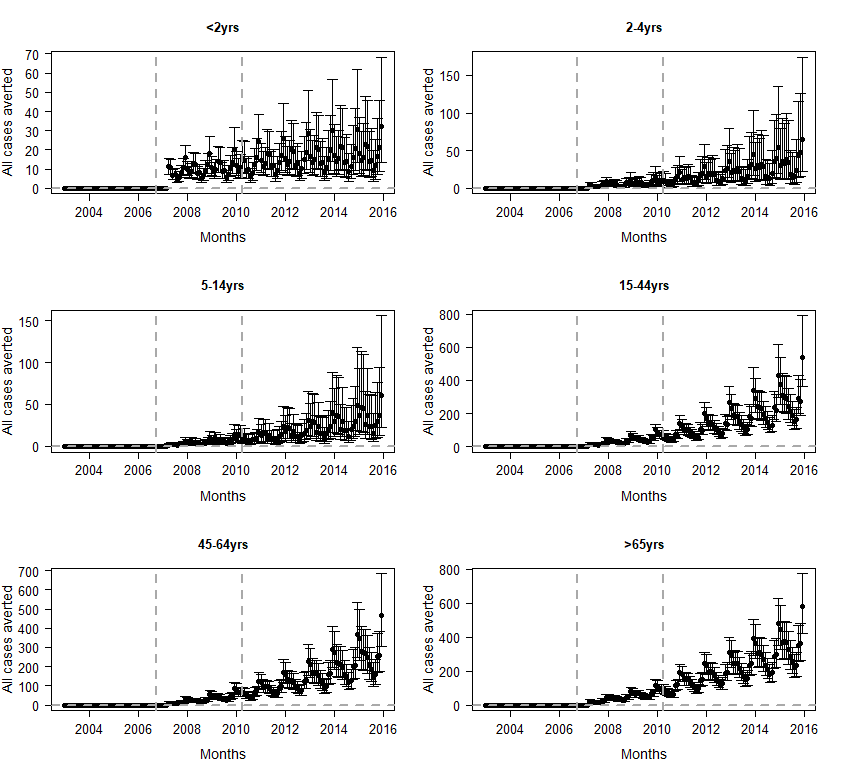


**Figure S9**. Estimated monthly pneumonia admissions averted due to the introduction of the vaccination programmes by age category. Error bars represent the 95% credible intervals. Vertical lines represent the months when PCV7 and PCV13 were introduced into the immunisation programme, respectively.

### Combined age groups

We also combine the data for all age groups for each disease category. The median time trend, rate ratios over time and admission cases avoided for septicaemia, meningitis, pneumonia and all pneumococcal disease for all age groups combined are shown in Figure S10, S11 and S12, respectively.


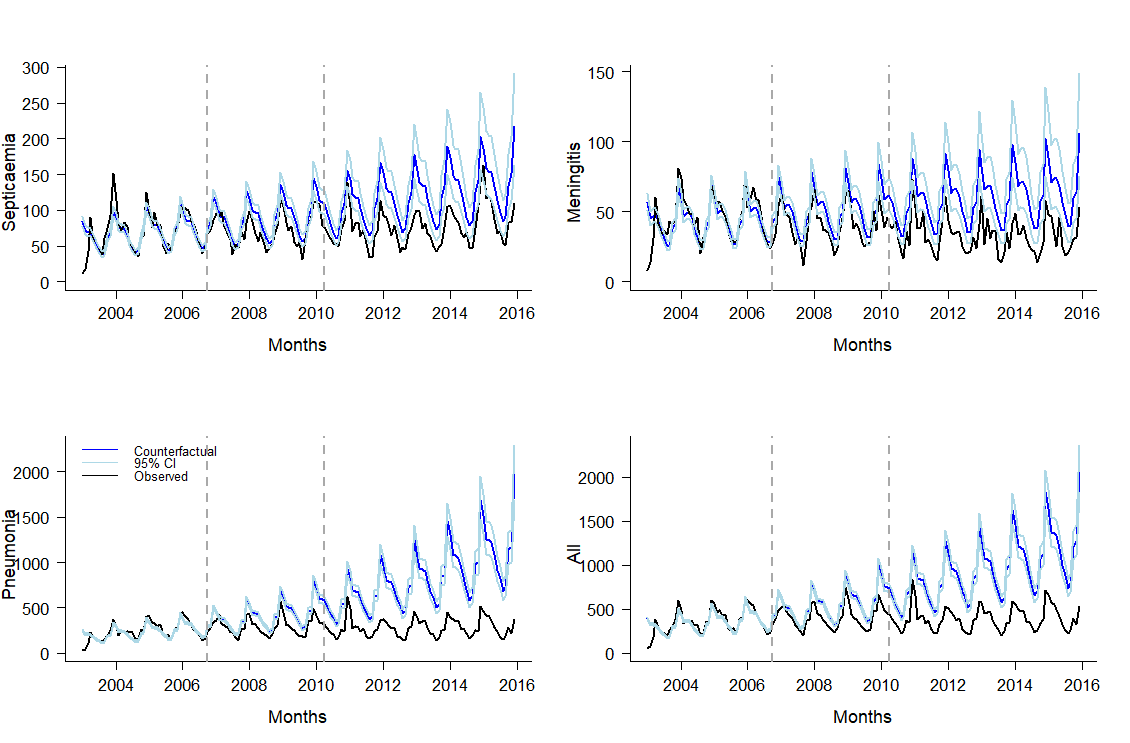


**Figure S10**. Observed and fitted monthly trends in septicaemia, meningitis, pneumonia and all pneumococcal disease hospitalisations between 2003 and 2015 by age category. Vertical grey lines represent the months when PCV7 and PCV13 were introduced into the immunisation programme, respectively.


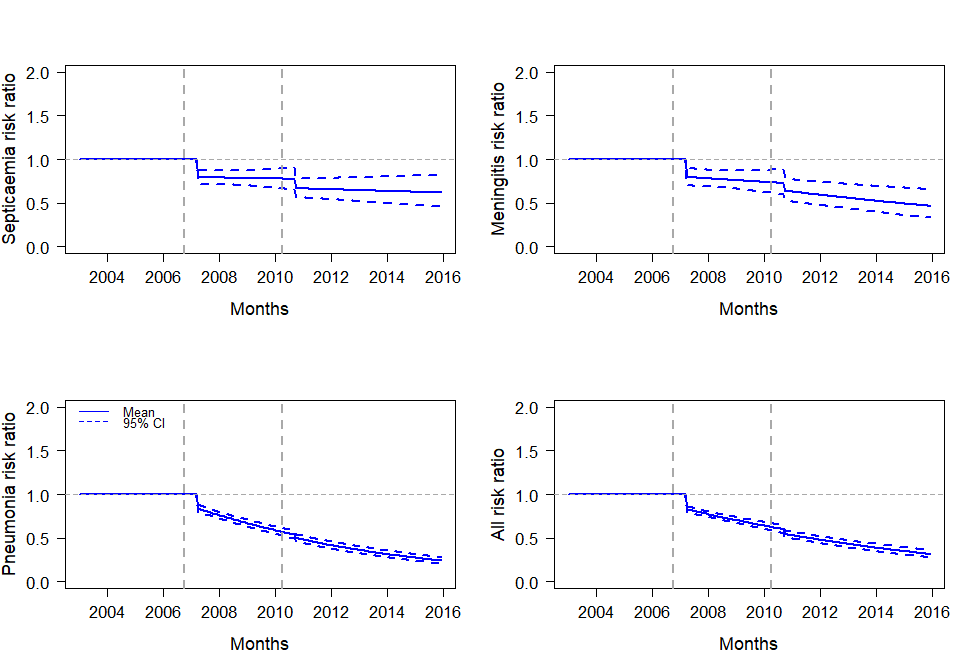


**Figure S11**. Septicaemia, meningitis, pneumonia and all pneumococcal disease monthly hospitalisation rate ratios over time by age category. Dashed lines represent the 95% credible intervals. Vertical grey lines represent the months when PCV7 and PCV13 were introduced into the immunisation programme, respectively.


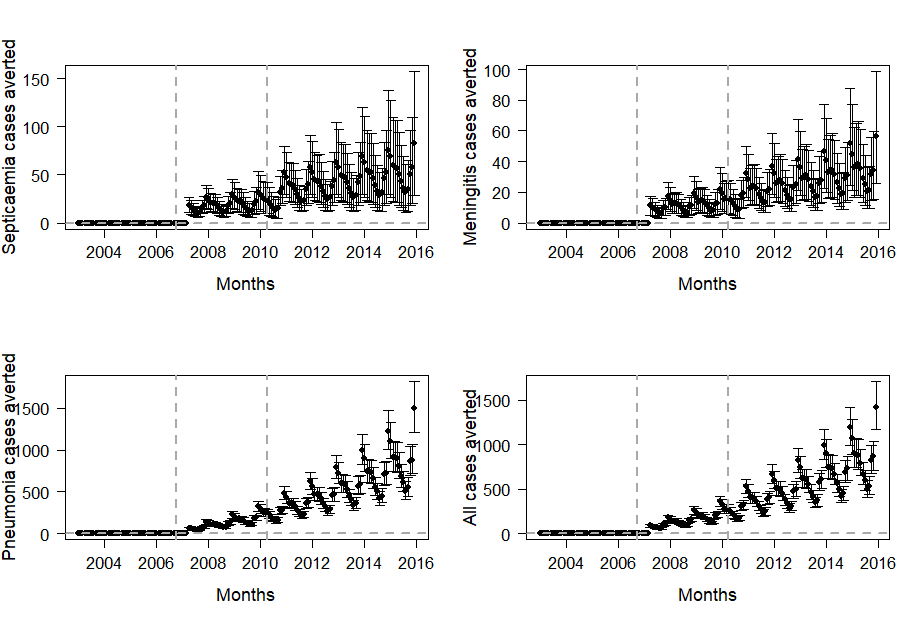


**Figure S12**. Estimated monthly septicaemia, meningitis, pneumonia and all pneumococcal disease admissions averted due to the introduction of the vaccination programmes by age category. Error bars represent the 95% credible intervals. Vertical lines represent the months when PCV7 and PCV13 were introduced into the immunisation programme, respectively.

# Sensitivity analysis

First, we fitted the three different models to different data sets. The interrupted time series is fitted to the whole data sets with dummy variables indicating change points. The no PCV13 counterfactual is fitted from 2003 to 2010 and all vaccines are switched off and lastly the no PCV counterfactual is fitted to the pre-PCV data spanning 2003 to 2006 and the extrapolated up to 2015.


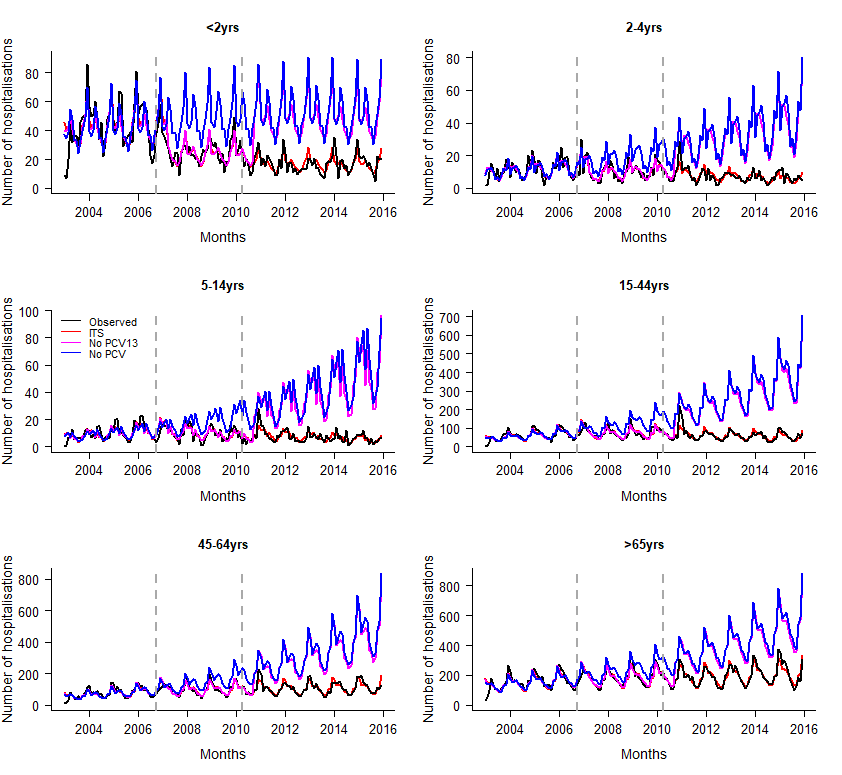


**Figure S13**. Observed and fitted monthly trends in all pneumococcal disease hospitalisations between 2003 and 2015 by age category. Vertical grey lines represent the months when PCV7 and PCV13 were introduced into the immunisation programme, respectively. Three different data sets were used three models.

Next, we also assume that the impact of PCV7 and PCV13 are similar and fit the interrupted time series with only one interrupted time point at the beginning of the PCV7 vaccination.


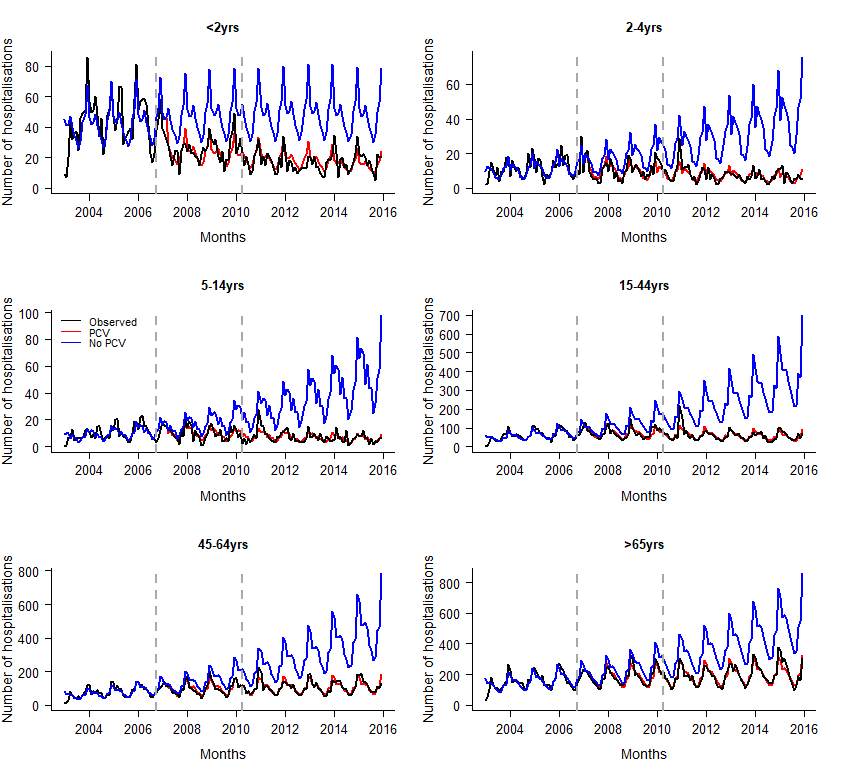


**Figure S14**. Observed and fitted monthly trends in pneumococcal disease hospitalisations between 2003 and 2015 by age category. Vertical grey lines represent the months when PCV7 and PCV13 were introduced into the immunisation programme, respectively. There was no distinction between PCV7 and PCV13 periods.

Next, we fit the counterfactual model using different datasets by adjusting the time when the vaccine’s effect is started.


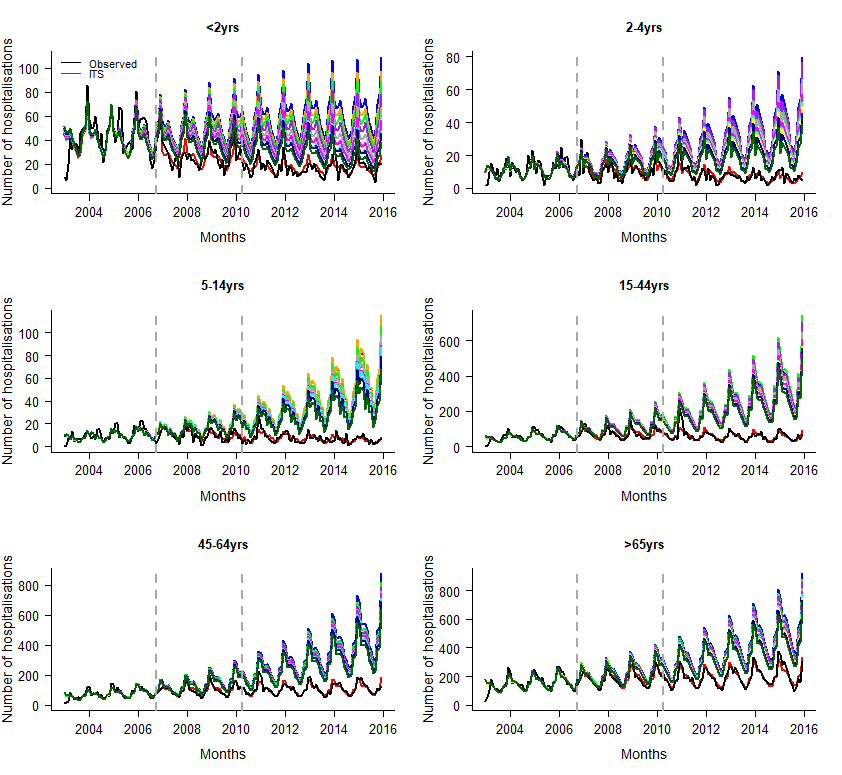


**Figure S15**. Observed and fitted monthly trends in septicaemia hospitalisations between 2003 and 2015 by age category, when there is a variation in lag period before any vaccine effect is expected (from October 2006 (blue line) to July 2007 (green line)). Vertical grey lines represent the months when PCV7 and PCV13 were introduced into the immunisation programme, respectively.


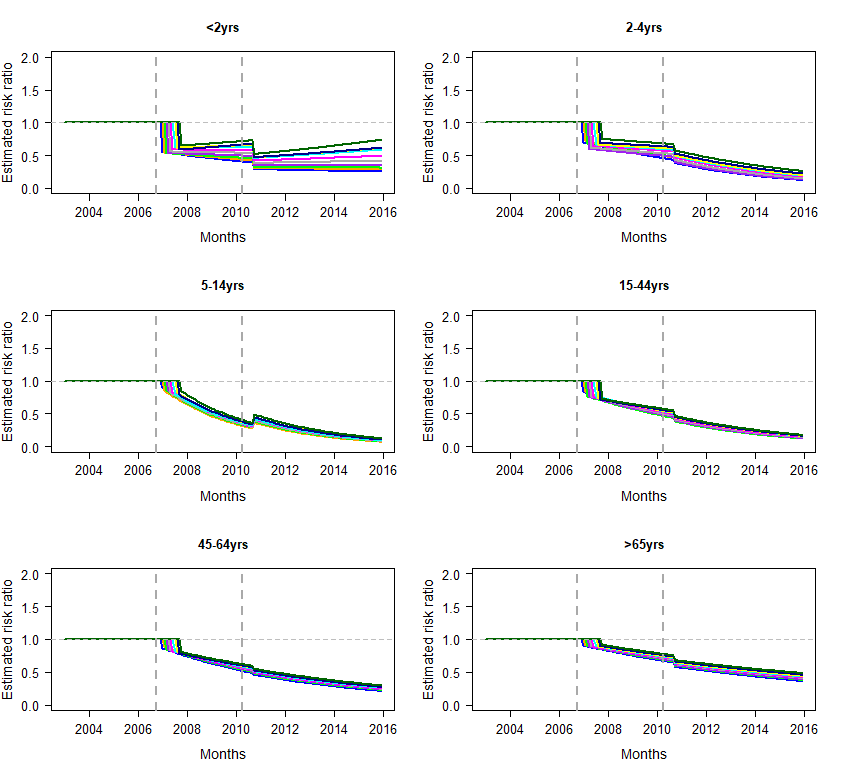


**Figure S16**. Estimated monthly risk ratios for pneumococcal disease hospitalisations between 2003 and 2015 by age category, when there is a variation in lag period before any vaccine effect is expected (from October 2006 (blue line) to July 2007(green line)). Vertical grey lines represent the months when PCV7 and PCV13 were introduced into the immunisation programme, respectively.

# References

1. Plummer M. JAGS: A program for analysis of Bayesian graphical models using Gibbs sampling. *Proceedings of DSC* 2003; **2**: 1.

2. Team RC. R: A Language and Environment for Statistical Computing. Vienna, Austria: R Foundation for Statistical Computing; 2014.
